# Development of Beta-Amyloid-Specific CAR-Tregs for the Treatment of Alzheimer’s Disease

**DOI:** 10.3390/cells12162115

**Published:** 2023-08-21

**Authors:** Valerie Saetzler, Tobias Riet, Andrea Schienke, Pierre Henschel, Kiara Freitag, Alexander Haake, Frank L. Heppner, Laura Elisa Buitrago-Molina, Fatih Noyan, Elmar Jaeckel, Matthias Hardtke-Wolenski

**Affiliations:** 1 Department of Gastroenterology, Hepatology, Infectious Diseases & Endocrinology, Hannover Medical School, 30625 Hannover, Germany; saetzler.valerie@mh-hannover.de (V.S.); tobias.riet@netcologne.de (T.R.); schienke.andrea@mh-hannover.de (A.S.); henschel.pierre@mh-hannover.de (P.H.); buitrago.laura@mh-hannover.de (L.E.B.-M.); noyan.fatih@mh-hannover.de (F.N.); jaeckel.elmar@mh-hannover.de (E.J.); 2 Department I of Internal Medicine, Tumor Genetics, University Hospital of Cologne and Center for Molecular Medicine Cologne (CMMC), University of Cologne, 50931 Cologne, Germany; 3 Department of Neuropathology, Charité-Universitätsmedizin Berlin, Freie Universität Berlin and Humboldt-Universität zu Berlin, 10117 Berlin, Germany; kiara.freitag@charite.de (K.F.); alexander.haake@charite.de (A.H.); frank.heppner@charite.de (F.L.H.); 4 German Center for Neurodegenerative Diseases (DZNE) within the Helmholtz Association, 10117 Berlin, Germany; 5 Department of Liver Transplantation, Multi Organ Transplant Program, University Health Network, University of Toronto, Toronto, ON M5T 0S8, Canada; 6 Institute of Medical Microbiology, University Hospital Essen, University Duisburg-Essen, 45147 Essen, Germany

**Keywords:** chimeric antigen receptor, Alzheimer’s disease, beta-amyloid, regulatory T cells, single-chain fragment

## Abstract

Background: Alzheimer’s disease (AD) is a neurodegenerative disease that remains uncured. Its pathogenesis is characterized by the formation of β-amyloid (Aβ) plaques. The use of antigen-specific regulatory T cells (Tregs) through adoptive transfer has shown promise for the treatment of many inflammatory diseases, although the effectiveness of polyspecific Tregs is limited. Obtaining a sufficient number of antigen-specific Tregs from patients remains challenging. Aims and Methods: To address this problem, we used an antibody-like single-chain variable fragment from a phage library and subsequently generated a chimeric antigen receptor (CAR) targeting β-amyloid. Results: The β-amyloid-specific CARs obtained were stimulated by both recombinant and membrane-bound Aβ isolated from the murine brain. The generated CAR-Tregs showed a normal Treg phenotype, were antigen-specific activatable, and had suppressive capacity. Conclusion: This study highlights the potential of CAR technology to generate antigen-specific Tregs and presents novel approaches for developing functional CARs.

## 1. Introduction

Alzheimer’s disease (AD) is a neurodegenerative disease affecting over 33 million people worldwide according to the World Health Organization (WHO). With increasing life expectancy, these numbers are expected to increase drastically, aggravating the disease in the future. The main symptoms are dementia, behavioral changes, and language problems, which not only affect the patients but also the people surrounding them.

The pathogenesis of AD is still not fully understood. However, it is thought to be a multifactorial disease involving genetics, environmental factors, neuroinflammation, and neuronal loss. It is marked by the formation of β-amyloid (Aβ) plaques and their extracellular deposition in the brain tissue and by the hyperphosphorylation of tau protein, leading to intracellular neurofibrillary tangles. Consequently, resident innate immune cells of the central nervous system (CNS) are activated to phagocytize neurotoxic deposits [1]. Although microglia are generally neuroprotective, they are altered in AD to promote neurodegeneration, leading to typical neuronal disease symptoms [2,3]. Thus, the role of microglia is likely to be more complex. Whether they are beneficial or detrimental to AD is debatable. Microglia respond to Aβ pathology by transitioning to disease-associated microglia, which are associated with alterations in their proliferation, phagocytosis, and inflammatory responses. For example, they can directly damage neuronal synapses or activate astrocytes via pro-inflammatory factors (Il-1α, TNFα, and C1q), which in turn destroy neurons and oligodendrocytes [4,5].

To date, there is no cure for AD; however, the first disease-modifying approach showed significant improvement in cognitive function in clinical trials and was approved in June 2021 by the U.S. Food and Drug Administration (FDA) for the treatment of mild and early AD (NCT02477800). This approach uses a human IgG1 anti-Aβ monoclonal antibody that induces the clearance of insoluble Aβ plaques by binding to these aggregates in a dose- and time-dependent manner [6]. This makes it the first drug for AD that not only treats the symptoms but also reduces Aβ plaques in the brain. However, it has not been tested in later stages or in more severe forms of the disease, which affect at least 50% of AD patients, leaving them without any disease-modifying treatment (DMT) option [7,8].

Microglia are usually controlled by CNS-resident CD4^+^ CD25^+^ Forkhead box P3 (Foxp3)^+^ regulatory T cells (Tregs), which play an important role in the maintenance of immune tolerance and homeostasis. In healthy individuals, Tregs can contain microglia-mediated neuroinflammation via soluble immunosuppressive factors, such as IL-10, TGF-β, or IL-35, but their function is compromised in patients with AD [2,9]. The neuroprotective role of Tregs has been studied extensively in AD mouse models, and Faridar et al. showed that ex vivo expansion of Tregs can restore their immunosuppressive functions in patients with AD [2,10,11]. Studies that show no beneficial or detrimental effects of Tregs have argued that this effect is highly debated. However, polyclonal Treg therapy is limited by the need for high cell numbers to achieve significant effects. In addition, non-specific immunosuppression by polyspecific Tregs may result in potential side effects, such as opportunistic infections and increased tumor risk [12].

Hence, the employment of antigen-specific Tregs is enticing, as they are known to be polyclonal Tregs in many different (auto-)inflammatory disease settings (e.g., type 1 diabetes, multiple sclerosis, or skin transplantation). Moreover, they can dampen inflammation more efficiently in mouse models [13,14,15,16]. We achieved antigen specificity by transducing Tregs with a chimeric antigen receptor (CAR), converting them into CAR-Tregs. The advantages of CAR-Tregs over (TCR-)Tregs are that they are not MHC restricted and that they can induce local and specific tolerance to their target tissue in different diseases [17]. The ability to be activated in a tissue-specific manner is conferred on CAR-Tregs by a single-chain variable fragment (scFv), which forms the antigen-binding domain of CAR. Therefore, screening for scFvs with high affinity for a disease-specific target (in this case, Aβ) is one of the most important steps during CAR development.

In this study, we present a new strategy for obtaining Aβ-specific scFvs. Subsequently, the generated Aβ-specific CAR-Tregs were stable and functional in vitro. This opens up the possibility of using them in preclinical models or therapeutic approaches for AD, even in later and more severe stages of the disease.

## 2. Materials and Methods

### 2.1. Generation of the CAR

The binding scFv PaD172-F12 was cloned into a second-generation CAR construct consisting of a murine CD8 hinge and transmembrane domain. Intracellular signalling was ensured via a CD28 costimulatory and CD3ζ activation domain. A γ-retroviral RSF91 expression vector was used to express CAR. Furthermore, transduction with this construct led to the expression of the transcription factor Foxp3 and the reporter gene Thy1.1 (CD90.1). 

### 2.2. Brain Lysates

For the brain lysates, the entire hemispheres of mouse brains were homogenized in Tris-buffered saline (TBS) buffer (20 mM Tris, 137 mM NaCl, pH 7.6) with added proteinase inhibitors using a tissue homogenizer and a 1 mL syringe with G26 cannulas. After centrifugation at 100,000× *g* for 45 min at 4 °C, the supernatant containing soluble Aβ was collected, and it is referred to in the following as “TBS fraction”. For the “TX fraction”, the pellet was resuspended in 1% triton x-100 in TBS and centrifuged again (100,000× *g*, 45 min, 4 °C). After this step, the supernatant contained the membrane-bound Aβ. To retrieve insoluble Aβ, the pellet was resuspended in 2% SDS in water and centrifuged (100,000× *g* for 45 min at 4 °C). The so-called “SDS fraction” can be found in the supernatant. All brain lysate fractions were snap frozen in liquid nitrogen upon collection and stored at −80 °C until needed. Protein concentrations were determined using the QuantiPro BCA Protein Assay Kit (Pierce, Schwerte, Germany), according to the manufacturer’s protocol.

### 2.3. Production of Retroviral Particles 

HEK-293T cells were transfected with K73 (*env*), 5 G/P (*gag/pol*), and the CAR construct using PEIpro^TM^ (Polyplus, Illkirch, France) according to the manufacturer’s protocol. Retroviral particles produced in the supernatant were collected at 24 and 32 h after transfection. The collected medium (DMEM with 10% heat-inactivated FCS, 1% penicillin/streptomycin) was filtered (0.22 µm) and centrifuged at 10,000× *g* overnight at 4 °C using an ultracentrifuge. The next morning, the medium was discarded, and the pellets were resuspended in fresh medium and stored at −80 °C.

### 2.4. Flow Cytometry Staining

For flow cytometric analysis of surface proteins, 1 × 10^6^ cells were stained with a 1:100 antibody diluted in PBS for 20 min at 4 °C. The following antibodies were used: anti-human IgG-F(ab’)2 (polyclonal, Jackson ImmunoResearch, Ely, UK), anti-CD69 (H1.2F3, Becton Dickinson, Heidelberg, Germany), anti-CD127 (SB/199, BioLegend, Koblenz, Germany), anti-CD62L (MEL-14; BioLegend), anti-CTLA4 (UC10-4B9, BioLegend), anti-GITR (DTA-1, eBioscience, Frankfurt, Germany), anti-CD25 (PC61, Becton Dickinson), anti-CD4 (RM4-5, BioLegend), anti-Thy1.1 (OX-7, BioLegend), and anti-CD8a (53-6.7; BioLegend). Fixable viability dye (eBioscience) was used. For intracellular staining, a fixation/permeabilization kit (eBioscience) and anti-Foxp3 (MF-14, BioLegend) antibodies were used (1:100, 30 min, RT).

For phenotypic analysis, a fluorescence minus one (FMO) control was performed for each marker. After measurement with the flow cytometer, cells were gated either on their Thy1.1 (for cTregs) or on their CD4 expression (nTregs). The histograms were normalized to the mode.

### 2.5. NFAT-GFP Assay

This reporter cell line was kindly provided by Prof. Dr. L. Klein from the Ludwig Maximilian University, Munich, Germany. First, NFAT-GFP cells were retrovirally transduced with the CAR construct via spininoculation (850× *g*, 37 °C, 1 h, MOI: 5) using protamine sulfate (4 μg/mL, Sigma-Aldrich, Steinheim am Albuch, Germany). Some cells were left untransduced and were used as controls. Then, 48 h after transduction, NFAT-GFP cells were incubated with different stimuli for another 24 h: PBS, plate-bound human [Gly22]-β-Amyloid (1-42) protein (Arctic Mutation, Eurogentec, Liège, Belgium) (250 ng), or brain lysates (500 ng total protein). Cells were analyzed by flow cytometry for their transduction efficiency (anti-IgG-F(ab’)2) and CAR activation (GFP expression).

### 2.6. nTreg and CD8 T Effector Cell Isolation 

Murine splenocytes were isolated and stained with the anti-CD4, anti-CD8, and anti-CD25 antibodies. Cells were purified using a cell sorter (FACSAria II, Becton Dickinson) and gated on CD4^-^CD8^+^ or CD8^-^CD4^+^CD25^+^ cells. FACS was performed at the cell sorting facility of the Hannover Medical School. 

### 2.7. Production of CAR-cTregs

Murine splenocytes were isolated and CD4^+^ cells were magnetically enriched using a MojoSort Mouse CD4 T Cell Isolation Kit (BioLegend). The cells were then cultured in Treg medium (complete RPMI supplemented with 1% Glutamax, 10% heat-inactivated FCS, 2% HEPES, 1% nonessential amino acids, 1% sodium pyruvate, 0.1% β-mercaptoethanol, and 1% penicillin/streptomycin) containing 500 IU IL-2/mL (Proleukin S, Novartis, Basel, Switzerland) and anti-CD3/CD28-beads (1:1) (Treg Expansion Kit, Miltenyi Biotec, Bergisch Gladbach, Germany) for cell activation. After 48 h, the cells were retrovirally transduced with the CAR construct via spin inoculation (850× *g*, 37 °C, 1 h, MOI:5) using protamine sulfate (4 μg/mL, Sigma-Aldrich). Another 48 h later, the cells were de-beaded using a MACSiMAG™ Separator (Miltenyi Biotec) and used for different assays.

### 2.8. Activation Assay

CD4^+^ cells and CAR-cTregs were incubated overnight in Treg medium containing 20 IU IL-2/mL. The next day, their activation status was measured using flow cytometric analysis of CD69. The cells were then incubated with plate-bound human protein (250 ng) or PBS controls for 24 h before analyzing the activation status (anti-CD69) and transduction efficiency using flow cytometry (anti-Thy1.1).

### 2.9. Suppression Assay

Isolated CD8^+^ cells (Teffs) were stimulated overnight with anti-CD3/CD28 beads. The following day, the cells were de-beaded, labeled with 5 mM CFSE (CellTrace™ CFSE Cell Proliferation Kit, Thermo Fisher Scientific, Braunschweig, Germany), and co-cultured at different ratios with either nTregs or CAR-cTregs for 5 days. Every two days, the cells received fresh Treg medium containing 500 IU IL-2/mL. To assess the functionality of different Treg types, the mean fluorescence intensity (MFI) of CFSE in the Teffs was measured using flow cytometry as an indicator of cell proliferation. Teffs without co-cultured Tregs were used as the controls. 

### 2.10. Statistical Analysis

Statistical analyses were performed using GraphPad Prism software (version 8, GraphPad Software, La Jolla, CA, USA). One-way ANOVA with Tukey’s multiple comparison test was performed. Significance was defined as *, **, ***, and ****, which indicate values of *p* < 0.05, *p* < 0.01, *p* < 0.001, and *p* < 0.0001, respectively.

### 2.11. Software

Flow cytometric data were analyzed using FlowJo software (version 10, Becton Dickinson). Schematic depictions were created using Biorender.com and figures were created using Microsoft PowerPoint.

## 3. Results

### 3.1. Generation of the Beta-Amyloid-Specific CAR

Beta-amyloid peptides (Aβ^1−42^) are produced during the pathogenesis of Alzheimer’s disease, but, at the same time, the ability to clear them efficiently from the brain is impaired. This results in monomeric peptides self-aggregating into oligomers and, eventually, into insoluble fibrils known as Aβ plaques. These plaques and their oligomeric form are considered toxic forms of Aβ, and their extracellular deposition leads, among other factors, to Alzheimer’s disease.

Therefore, we aimed to generate CAR-Tregs that can recognize these plaques and counteract this disease.

**Figure 1:**
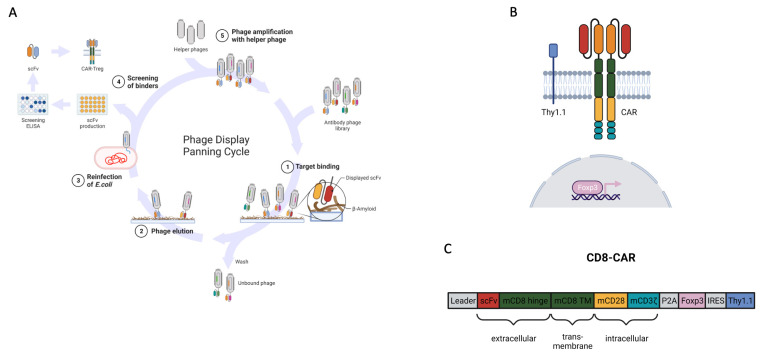
Protein panning as a tool to generate specific scFv used for CARs (**A**) Overview of panning protocol on plate-bound antigen showing one complete panning round. (**B**) Scheme of murine 2nd generation CAR containing scFv with additional Foxp3 and Thy1.1 as reporter. (**C**) Genetic construct of murine 2nd generation short-hinge (CD8-based) CAR containing scFv, Foxp3, and Thy1.1 as reporter gene under control of IRES.

To engineer CARs, an antigen-binding domain in the form of a single-chain fragment (scFv) is required. ScFvs, which were used as antigen receptors in our CAR constructs, were screened using phage display from human naïve antibody HAL7/8 gene libraries, as previously described (Figure 1A) [18]. DNA sequences of the positive candidates were isolated, sequenced, and cloned into a second generation CD28/CD3ζ CAR scaffold with a CD8-derived hinge (Figure 1B,C) [19]. We assembled a beta-amyloid-specific CD8-CAR from clone PaD172-F12.

### 3.2. Beta-Amyloid-Specific CAR Can Be Activated by Human Peptide

To further investigate the activating potential of Aβ-specific scFvs, PaD172-F12 was tested in CAR format.

The CAR construct was transduced into a murine NFAT-GFP reporter T cell line that encoded GFP under the control of the NFAT-dependent minimal IL-2 promoter [15,19]. Upon activation of these cells by CAR stimulation of the respective target structure, upregulation of NFAT leads to GFP expression.

**Figure 2:**
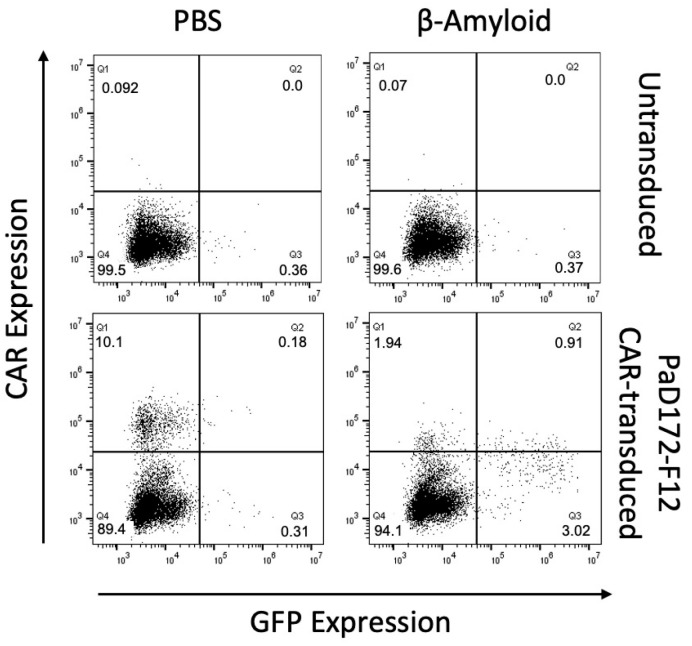
Beta-amyloid-specific CAR can be activated by human peptide. CAR activation in murine T cell line following stimulation by target antigen. NFAT activation is reported by GFP expression, and anti-Fab antibody was used for CAR staining. CARs were tested with β-amyloid (**right blots**) or medium control (PBS, **left blots**).

Therefore, CAR-transduced cells were co-cultured with immobilized Aβ^1−42^ peptide. The retroviral CAR transduction efficiency was approximately 10%, as indicated by CAR staining in the negative control (Figure 2). Autoactivation in this unstimulated sample was minimal, with <0.5% GFP-positive cells. In contrast, the target peptide activated the cells bearing the CAR construct. CAR activation was observed in 4% of the cells, corresponding to 40% of the cells expressing CAR.

Thus, it can be concluded that Aβ-specific CAR can be stimulated by Aβ^1−42^ peptide in a murine T cell line.

### 3.3. Murine Brain Lysates Contain Membrane-Bound Ab Plaques That Can Activate CARs

In addition, Aβ-specific CAR was tested on different brain lysate fractions from wild-type mice (wt) and the AD-like mouse model APPPS1 carrying transgenes for the human amyloid precursor protein (APP) bearing the Swedish mutation as well as a presenilin 1 mutation. APPPS1 transgenic mice develop strong Aβ pathology, including soluble Aβ species and Aβ plaques. After consecutive protein extractions of the brain homogenates, Aβ was separated into three fractions based on solubility. The TBS fraction contained soluble Aβ_1–40_ and Aβ_1–42_, the TX fraction contained membrane-bound Aβ_1–40_ and Aβ_1–42_, and insoluble Aβ_1–40_ and Aβ_1–42_ were isolated from the SDS fraction.

**Figure 3:**
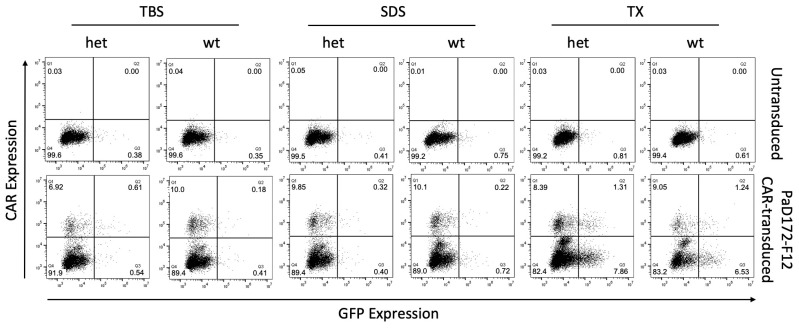
Murine brain lysates containing membrane-bound Aβ can activate CARs. NFAT activation is reported by GFP expression, and anti-Fab antibody was used for CAR staining. A non-transduced (**upper row**) or PaD172-F12 CAR-transduced (**lower row**) murine T cell line was tested with the three lysate fractions containing soluble Aβ (TBS), insoluble Aβ (SDS), or membrane-bound Aβ (TX). These fractions were either generated from APPPS1 (het) or wild-type (wt) mice.

The two lysate fractions containing soluble Aβ (TBS) or insoluble Aβ (SDS) did not activate Aβ-specific CAR in the reporter T cell line from either APPPS1 or wild-type mice (Figure 3). In contrast, the lysate fraction containing membrane-bound Aβ (TX) was able to activate approximately 50% of the Aβ-specific CAR^+^ cells. However, it was not determined whether this fraction was obtained from wild-type or APPPS1 animals. It is worth noting that it is a well-known phenomenon that some CAR cells have less CAR on their surface after antigen contact and activation, which is why most cells were found in the lower right quadrant of the dot plots.

In summary, the murine Aβ-specific CAR reporter T cell line can be stimulated by both the Aβ^1−42^ peptide and membrane-bound Aβ, but not by soluble or insoluble Aβ.

### 3.4. Aβ-Specific Converted CAR-Tregs Do Not Differ in Phenotype from Natural Tregs

Because murine natural Tregs (nTregs) expand poorly compared to human Tregs, converted Tregs (cTregs) were used for subsequent experiments. After transduction with the CAR construct (Figure 1B), isolated CD4^+^ T cells from splenocytes of BALB/c mice received an additional exogenous Foxp3 gene. This process converts these cells into stable Tregs [19,20,21].

**Figure 4:**
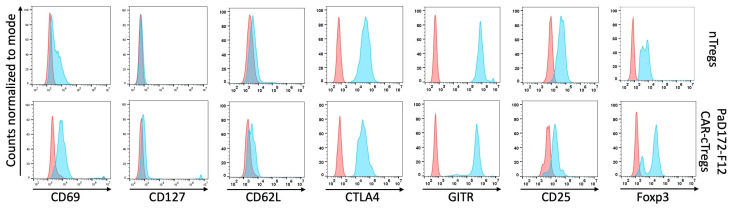
Phenotype of CAR-cTregs equals nTregs. For phenotype analysis, CD4^+^ T cells were transduced with a CAR construct including the Foxp3 sequence; nTregs were sorted for CD4^+^CD25^high^. Expression of CD69, CD127, CD62L, CTLA-4, GITR, CD25, and Foxp3 is shown. Fluorescence minus one staining served as a control.

These stable Tregs differed slightly from nTregs in terms of CD25, CD62L, CD127, CTLA-4, GITR, and Foxp3 expression (Figure 4). Only Helios was not expressed by cTregs, because only thymic-derived Tregs expressed this transcription factor (data not shown). The expression of CD69 in CAR-cTregs was slightly higher than that in nTregs, because it is an early activation marker and T cells need to be stimulated before they can be transduced with CAR constructs, whereas nTregs were freshly isolated.

In summary, CAR-cTregs phenotypically look like nTregs.

### 3.5. Primary Murine Aβ-Specific cTregs Can Be Activated by Aβ-Peptide

Because the previous results were obtained with a T cell line, it is of great interest to determine whether primary cells can be activated in the same way.

**Figure 5:**
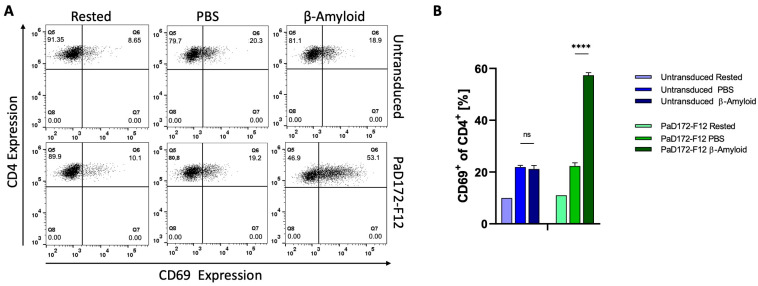
Aβ-specific CAR-Tregs are activated by β-Amyloid. (**A**) Murine T cells were transduced with 2nd generation short-hinge (CD8-based) CAR containing β-amyloid-specific scFv binder PaD172-F12, Foxp3, and Thy1.1 for generating CAR-Tregs. Flow cytometric CD69 expression is shown for CD4+ pre-gated on Thy1.1^+^ rested, after stimulation with β-Amyloid, or without simulation (PBS). (**B**) β-Amyloid-specific CAR-Tregs specifically upregulated CD69 after stimulation with β-Amyloid (right, green bar) in comparison to unstimulated cells (middle green bar), rested cells (left green bar), and non-transduced control cells (blue bars). Data are presented as mean ± SD (*n* = 3). *p* values are determined by two-way ANOVA and multiple comparison testing (ns = not significant).

For this purpose, Aβ-specific CAR cTregs were analyzed. Non-transduced CD4^+^ T cells and rested CAR-cTregs expressed approximately 10% of the background of the activation marker CD69 (Figure 5). The background increased to 20% in both populations when the cells were incubated on PBS-coated plates. However, if the non-transduced CD4 T cells or CAR-cTregs are stimulated with the Aβ^1−42^ peptide, activation of more than 50% occurs only in the Aβ-specific CAR-cTreg population.

Thus, it can be concluded that Aβ-specific CAR can be stimulated by the Aβ^1−42^ peptide in murine cTregs.

### 3.6. Aβ-Specific CAR-Tregs Can Inhibit Activated CD8^+^ T Cells

The decisive parameter for Treg efficiency is the inhibition of T effector cells.

**Figure 6:**
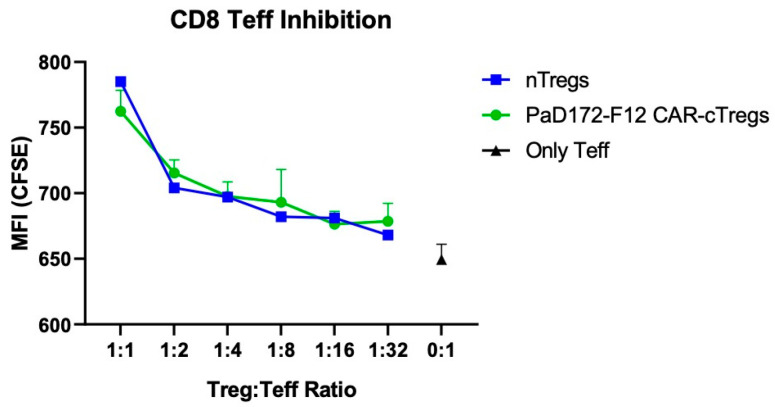
Aβ-specific CAR-cTregs showed the same suppressive capacity as nTregs.

Therefore, naïve CD8^+^ T cells from splenocytes were stained for the proliferation marker, CFSE. Subsequently, CD8 effectors (Teff) were activated overnight with anti-CD3/CD28 activation beads. Cell proliferation caused the CFSE signal to decrease to an MFI of 650 (Figure 6). When nTregs or Aβ-specific CAR-Tregs were added, proliferation decreased sharply, depending on the number of Tregs. Inhibition was similar in both Treg populations.

In conclusion, Aβ-specific CAR-cTregs were as functional as nTregs in inhibiting activated T cells.

CD8 effectors (Teff) were activated using anti-CD3/CD28 activation beads and labeled with CFSE. Without Treg inhibition (0:1), MFI was defined as basic proliferation (black dot). Both nTregs (blue dots) and Aβ-specific CAR-Tregs (green dots) were added at indicated ratios.

## 4. Discussion

We generated CAR against beta-amyloid and subsequently engineered CAR cTregs. The resulting Aβ-specific CAR-cTregs displayed a normal Treg phenotype, could be activated in vitro, and could suppress activated T cells. These results describe a new, potentially therapeutically useful strategy to generate large numbers of antigen-specific Tregs by changing the specificity of T cells and converting them into Tregs. This protocol can be performed with each donor and has a limited in vitro culture time and, therefore, off-the-shelf capacity [19,20,21,22,23]. Moreover, cTregs exhibited a normal proliferative capacity and were highly responsive to antigen stimulation [19,20,21].

Regulatory T cells play an important role in maintaining tolerance, and impairment of their suppressive function leads to the development of autoimmune diseases [24]. Owing to their ability to balance immune responses, they have been used for the treatment of diverse autoimmune disorders in a clinical setting [25,26]. Similarly, some neurological diseases, such as ALS or AD, are also characterized by neuroinflammation that cannot be contained by the Tregs of the patient [2,9,27]. However, the transfer of polyclonal Tregs improves the disease course in these patients [28].

Furthermore, antigen-specific Tregs have been shown to be superior to polyclonal Tregs in mouse models of autoimmunity, such as EAE for multiple sclerosis [14]. Chimeric antigen receptors can be used to confer antigen specificity on these cells. The successful use of CARs in the field of oncology is a promising outlook for immunotherapeutic strategies to treat autoimmune diseases using this technology. One advantage of CARs is that they are not restricted to the MHC molecules. This allows for specific signaling upon target recognition, and it can induce local immunosuppression if CAR-Tregs are used [29].

As the antigen-binding domain of CAR, scFvs must be carefully selected based on their capacity to specifically bind to the target protein. Different methods can be used to generate scFvs; for example, by generating hybridoma cells from antigen-immunized animals [30]. However, in this project, scFv from a phage display approach with human antibody libraries towards the target of interest was used [18,31].

The choice of the target is one of the most important steps in the generation of CARs. The impact of this decision was shown by Radichev et al., who laboriously generated and tested a functional CAR for the treatment of type 1 diabetes before realizing that their chosen target antigen was highly expressed on Tregs, rendering their CARs unusable [32]. Generally, the chosen target influences the efficacy of CAR-T cells, their homing ability, and possible side effects, such as off-target effects or target escape, as observed in patients treated with CAR-T cells [33,34]. However, there is not much data on what exactly defines a good target structure for CARs in an autoimmune setting, although some features are indispensable. These include high expression of the target in the disease-relevant organ with low or no expression in irrelevant organs, relevance or upregulation of the protein in the disease, and accessibility of the protein by CAR-Tregs.

Although the 4-1BB co-stimulatory domain can be used for CAR-T cells, it was shown that this domain is not as potent as the CD28 domain in Tregs. However, studies assessing the influence of costimulatory domains on the suppressive activity of CAR-Tregs are controversial. While Boroughs et al. demonstrated the superiority of CD28 domains, Koristka et al. observed no difference between the two domains in their capacity for suppression [35,36]. A possible reason why CD28 might be better for use in CAR-Tregs is that it plays an important role in Treg expansion and development [37]. CAR-T cell exhaustion and in vivo persistence are largely dependent on the costimulatory domain [38]. Some studies have demonstrated that 4-1BB is superior to the CD28 costimulatory domain in terms of CAR-T cell survival in vivo, which could also be the case for CAR-Tregs [39].

Although AD is not primarily an autoimmune disease, its emerging symptoms are due to neuroinflammation. The pathogenesis is thereby confined to the CNS, which is why treatment with CNS-specific CAR-Tregs is a feasible approach to contain local inflammation. Aβ plaques are hallmarks of AD, rendering them suitable targets for CAR. These neurotoxic aggregates are deposited extracellularly, to which CAR constructs preferentially bind to Aβ monomers.

For gene transfer of CAR constructs into different cell types, a γ-retroviral RSF91 expression vector was used. Other methods of cell modification, with which the CARs could be transferred, are, for example, lentiviral vector systems, endonucleases (e.g., CRISPR/Cas9 system), transposons (e.g., “sleeping beauty” system), or even mRNA technology for in vivo production of the CARs [40,41,42]. The γ-retroviral vector system is an FDA-approved method for CAR-T cell generation [43]. In addition to CAR, the expression vector also encodes a Foxp3 and Thy1.1 cassette [20,21]. These proteins are separated by the P2A-site and IRES, respectively. Because of the 2A self-cleaving peptide, Foxp3 was separated from the CAR construct during translation. However, this process is not fully efficient, leading to a nonlinear correlation between CAR and Foxp3 expression, which is why only the Thy1.1^high^ population can be used for therapeutic experiments to ensure a suppressive phenotype. Loss of expression efficiency in polycistronic vectors is a well-known phenomenon [44]. In contrast, the expression of Thy1.1 and CAR was similarly high, indicating that the IRES worked in this case more efficiently than the P2A-site. IRES uses a cap-independent mechanism to recruit the 40S ribosomal subunit and initiate translation. Therefore, it is independent of CAR expression [45]. However, IRES (582 bp) is significantly larger than 2A-peptides (~60 bp), which can pose a challenge for transduction with retroviral vectors. For efficient gene transfer, the vector should not exceed much more than 8–9 kb, which must be considered in the composition of CAR constructs.

However, it can be observed that the activation pattern of the CAR with this scFv was different between the types of activation. While they appear “normal” for stimulation with plate-bound protein, CAR expression was downregulated after activation with brain-derived Aβ plaques. Although receptor recycling is a normal process, it has been shown that some CARs are ubiquitinated and degraded in lysosomes [46]. Therefore, CAR takes longer to be expressed on the cell surface. This raises the question of whether CARs that exhibit internalization function as efficiently as CARs that do not. It has been demonstrated that this phenomenon is beneficial for the CAR function in T cells [47]. However, the exact opposite was shown in a different study, where CAR-Ts that internalized CAR were less efficient in their tumor-killing capacity [48]. To prevent ubiquitination, which leads to degradation, Li et al. developed a strategy in which all cytoplasmic lysine residues were mutated to arginines, leading to faster recycling of the receptors and more potent CAR-Ts [46]. In this assay, however, it is not clear why this phenomenon is only observed for some CAR constructs, while others have unchanged CAR expression after receptor activation.

## 5. Conclusions

We successfully created CAR-targeting beta-amyloid and subsequently developed CAR-cTregs. CAR-cTregs displayed typical Treg characteristics, could be activated, and demonstrated suppressive ability. In addition, cTregs maintained their normal ability to proliferate and displayed heightened responsiveness to antigen stimulation. These findings present a novel approach with a potential therapeutic value for generating abundant antigen-specific Tregs by modifying T cells with CARs and converting them into Tregs. This technique can be applied to any donor with a short in vitro culture period, making it readily available.

## Data Availability

The data presented in this study are available upon request from the corresponding author.
